# Diffusion tensor imaging and tractwise fractional anisotropy statistics: quantitative analysis in white matter pathology

**DOI:** 10.1186/1475-925X-6-42

**Published:** 2007-11-09

**Authors:** Hans-Peter Mueller, Alexander Unrath, Anne D Sperfeld, Albert C Ludolph, Axel Riecker, Jan Kassubek

**Affiliations:** 1 Department of Neurology, University of Ulm, Ulm, Germany

## Abstract

**Background:**

Information on anatomical connectivity in the brain by measurements of the diffusion of water in white matter tracts lead to quantification of local tract directionality and integrity.

**Methods:**

The combination of connectivity mapping (fibre tracking, FT) with quantitative diffusion fractional anisotropy (FA) mapping resulted in the approach of results based on group-averaged data, named tractwise FA statistics (TFAS). The task of this study was to apply these methods to group-averaged data from different subjects to quantify differences between normal subjects and subjects with defined alterations of the corpus callosum (CC).

**Results:**

TFAS exhibited a significant FA reduction especially in the CC, in agreement with region of interest (ROI)-based analyses.

**Conclusion:**

In summary, the applicability of the TFAS approach to diffusion tensor imaging studies of normal and pathologically altered brains was demonstrated.

## 1. Background

Diffusion tensor magnetic resonance imaging (DTI) is known to be an appropriate technique to map in vivo the diffusion in human brain white matter (WM). The directional dependence of diffusion in each voxel can be characterised by a 3 × 3 matrix called the diffusion tensor D→→
 MathType@MTEF@5@5@+=feaafiart1ev1aaatCvAUfKttLearuWrP9MDH5MBPbIqV92AaeXatLxBI9gBaebbnrfifHhDYfgasaacPC6xNi=xH8viVGI8Gi=hEeeu0xXdbba9frFj0xb9qqpG0dXdb9aspeI8k8fiI+fsY=rqGqVepae9pg0db9vqaiVgFr0xfr=xfr=xc9adbaqaaeGacaGaaiaabeqaaeqabiWaaaGcbaGafmiraqKbaSGbaSaaaaa@2D07@. The Eigenvectors and Eigenvalues of D→→
 MathType@MTEF@5@5@+=feaafiart1ev1aaatCvAUfKttLearuWrP9MDH5MBPbIqV92AaeXatLxBI9gBaebbnrfifHhDYfgasaacPC6xNi=xH8viVGI8Gi=hEeeu0xXdbba9frFj0xb9qqpG0dXdb9aspeI8k8fiI+fsY=rqGqVepae9pg0db9vqaiVgFr0xfr=xfr=xc9adbaqaaeGacaGaaiaabeqaaeqabiWaaaGcbaGafmiraqKbaSGbaSaaaaa@2D07@ reveal the diffusivity of water in each direction and therefore can be used to quantify the diffusivity by so-called fractional anisotropy (FA) maps on a voxelwise basis. This orientational information can also be the basis for the reconstruction of the interconnectivity of brain regions by following the pathways of the fibres. This technique is known as fibre tracking (FT). The basis of FT is to connect neighboured tensors consecutively along their principal directions. Many techniques referring to this topic have been published [1-5]. Most of them focus on qualitative imaging of the FT, 3-D visualisation and judgement by experienced operators. There have also been various efforts in using diffusion anisotropy as a marker for white matter tract integrity [6-8]. In these works, quantitative analysis has been performed by use of the underlying FA maps for selective statistics.

Smith et al. developed an algorithm for an alignment-invariant tract representation to overcome normalization problems; this approach is referred to as tract based spatial statistics (TBSS) [9-15]. In the present study, an analysis technique named tractwise fractional anisotropy statistics (TFAS) is presented. Hereby, bundles of FT are used in the sense of a skeleton, which is the basis of statistical analysis of the underlying FA maps. The novel character of TFAS is that it uses averaged DTI data sets, i.e. the processing steps of normalization and averaging are not performed on FA maps but on one newly created group averaged DTI data set. Based upon this data set, one FA map is calculated. The definition of the region under observation is consequently performed by anatomical connectivity. Whereas the connectivity of white matter regions is neither under investigation in region of interest (ROI)-based analysis methods nor in whole brain-based statistical analysis methods, TFAS was intended to analyse specific white matter regions as well as their connecting pathways not only in healthy brains, but also in distorted brain anatomy.

The prerequisite of statistical analysis at group level and arithmetic averaging of subject data is the normalisation to a standardised stereotactic space, e.g. the Montreal Neurological Institute (MNI) space. MNI defined a new standard brain by using a large series of MRI scans of normal controls, resulting in the MNI atlas [16]. MNI normalisation allows for arithmetic averaging of resulting FA maps.

FT with starting points in the corpus callosum (CC) was used to build skeletons for consecutive statistical analysis. The CC was chosen as the most appropriate structure in the brain since it is one of the white matter structures with an accumulation of mostly strongly directed fibres [17]. TFAS based on different skeletons was used to quantify interconnectivity and to map differences between patients with atrophy of the CC and age-matched healthy controls. As a model of CC alteration, patients with complicated hereditary spastic paraparesis (cHSP) were investigated. This rare neurodegenerative disease was chosen as a prototypical alteration across the whole structure of the CC. These patients' brains frequently show a thinned CC (tCC) [18,19]. Computer simulations that showed the validity of the applied MNI normalisation algorithms and the FT techniques complemented this work.

## 2. Methods

### 2.1. Data recording and subject population

DTI scanning protocols were performed on the same 1.5 *T *scanner (Symphony, Siemens Medical, Erlangen, Germany). Six healthy controls (3 men, 3 women, average age 32.7 ± 4.5), and 6 patients with (tCC) (3 men, 3 women, average age 32.5 ± 12.1) underwent the MRI protocol.

All DTI acquisitions consisted of 13 volumes (45 slices, 128 × 128 voxel, slice thickness 2.2 *mm*, in-plane voxel size 1.5 *mm *× 1.5 *mm*) representing 12 gradient directions and one scan with gradient 0 (*B*_0_). Echo time (TE) and repetition time (TR) were 93 *ms *and 8000 *ms*, respectively. *b *was 800 *s/mm*^2^, 5 scans were *k*-space averaged online by the Siemens SYNGO operating software. As a morphological background, a T_1_-weighted magnetisation-prepared rapid-acquisition gradient echo sequence was used (MPRAGE, TR = 9.7 *ms*, TE = 3.93 *ms*, flip angle 15°, matrix size 256 × 256 *mm*^2^, voxel size 1.0 × 0.96 × 0.96 *mm*^3^), consisting of 160–200 sagittal partitions depending on the head size.

### 2.2. Data processing

#### 2.2.1. Eddy current correction

Large discontinuities in bulk magnetic susceptibility produce local magnetic field gradients that notoriously degrade and distort DTI, particularly during echo-planar imaging [20]. These eddy current induced geometric distortions vary with the magnitude and direction of the diffusion sensitising gradients. For the correction of this distortion, the method proposed by Shen et al. [21] was applied. The technique relies on collecting pairs of images with reversed diffusion sensitising gradients – these paired images are distorted with eddy currents in opposite directions. A columnwise correction in the image domain along the phase encoding direction (anterior – posterior) was applied. This was performed by searching for the maximum value of the cross-correlation between two corresponding columns (of two paired volumes) while one is shifted and scaled (fitting routine: Simplex method [22]). Each column was then corrected by applying opposite shifts and scales equal to half of the correction. Other techniques for the eddy current correction were described in [23,24].

#### 2.2.2. Transformation to iso-voxels and smoothing

As the recording technique provided voxels with non-isotropic size (usually the slice thickness was larger than the in-plane voxel size), the DTI data sets were transformed into an isotropic grid with voxel size 1.0 × 1.0 × 1.0 *mm*^3 ^in the first step. The transformation chosen was a linear nearest neighbour transformation with

Itarg⁡et(i,j,k)=∑v=18avIv(l,m,n)
 MathType@MTEF@5@5@+=feaafiart1ev1aaatCvAUfKttLearuWrP9MDH5MBPbIqV92AaeXatLxBI9gBaebbnrfifHhDYfgasaacPC6xNi=xI8qiVKYPFjYdHaVhbbf9v8qqaqFr0xc9vqFj0dXdbba91qpepeI8k8fiI+fsY=rqGqVepae9pg0db9vqaiVgFr0xfr=xfr=xc9adbaqaaeGacaGaaiaabeqaaeqabiWaaaGcbaGaemysaK0aaSbaaSqaaiabdsha0jGbcggaHjabckhaYjabcEgaNjabdwgaLjabdsha0bqabaGccqGGOaakcqWGPbqAcqGGSaalcqWGQbGAcqGGSaalcqWGRbWAcqGGPaqkcqGH9aqpdaaeWaqaaiabdggaHnaaBaaaleaacqWG2bGDaeqaaOGaemysaK0aaSbaaSqaaiabdAha2bqabaGccqGGOaakcqWGSbaBcqGGSaalcqWGTbqBcqGGSaalcqWGUbGBcqGGPaqkaSqaaiabdAha2jabg2da9iabigdaXaqaaiabiIda4aqdcqGHris5aaaa@5206@

where *I*_*t *arg *et *_(*i*, *J*, *k*) was the voxel intensity at the new grid coordinates *i*, *j*, *k *and *l*, *m*, *n *were the original voxel coordinates in *x*, *y*, *z *direction, respectively. The factors *a*_*v *_were the 8 weighting factors for the interpolation.

Interpolation and smoothing are classical image processing problems for which variety of methods exist [25-27]. Mishra et al. [26] developed the idea of an anisotropic image interpolation method. Hereby, the kernel for interpolation was weighted by a factor depending on the local gradients. Thus, the sharpness of the image remained preserved. For smoothing, we chose a Gaussian kernel, i.e. a kernel sphere of radius 4 mm with Gaussian shape around each voxel. This kernel was weighted with the local gradients by

Itarg⁡et(i,j,k)=∑v=1NIv(l,m,n)/(rv+gtarg⁡et)∑v=1N1/(rv+gtarg⁡et)
 MathType@MTEF@5@5@+=feaafiart1ev1aaatCvAUfKttLearuWrP9MDH5MBPbIqV92AaeXatLxBI9gBaebbnrfifHhDYfgasaacPC6xNi=xI8qiVKYPFjYdHaVhbbf9v8qqaqFr0xc9vqFj0dXdbba91qpepeI8k8fiI+fsY=rqGqVepae9pg0db9vqaiVgFr0xfr=xfr=xc9adbaqaaeGacaGaaiaabeqaaeqabiWaaaGcbaGaemysaK0aaSbaaSqaaiabdsha0jGbcggaHjabckhaYjabcEgaNjabdwgaLjabdsha0bqabaGccqGGOaakcqWGPbqAcqGGSaalcqWGQbGAcqGGSaalcqWGRbWAcqGGPaqkcqGH9aqpdaWcaaqaamaaqadabaGaemysaK0aaSbaaSqaaiabdAha2bqabaGccqGGOaakcqWGSbaBcqGGSaalcqWGTbqBcqGGSaalcqWGUbGBcqGGPaqkaSqaaiabdAha2jabg2da9iabigdaXaqaaiabd6eaobqdcqGHris5aOGaei4la8IaeiikaGIaemOCai3aaSbaaSqaaiabdAha2bqabaGccqGHRaWkcqWGNbWzdaWgaaWcbaGaemiDaqNagiyyaeMaeiOCaiNaei4zaCMaemyzauMaemiDaqhabeaakiabcMcaPaqaamaaqadabaGaeGymaedaleaacqWG2bGDcqGH9aqpcqaIXaqmaeaacqWGobGta0GaeyyeIuoakiabc+caViabcIcaOiabdkhaYnaaBaaaleaacqWG2bGDaeqaaOGaey4kaSIaem4zaC2aaSbaaSqaaiabdsha0jGbcggaHjabckhaYjabcEgaNjabdwgaLjabdsha0bqabaGccqGGPaqkaaaaaa@77A1@

where *r*_*v *_was the distance between *I*_*t *arg *et *_and *I*_*v*_, and *g*_*t *arg *et *_was the absolute value of the gradient at position *(i,j,k)*. In this way, the local gradients weighted the interpolation kernel with a sharpness dependency.

### 2.2.3. Template generation and spatial normalisation

Spatial normalisation allowed for arithmetic averaging of the results obtained from different subjects in order to finally perform a comparison of groups of patients with certain disorders, e.g. neurodegenerative diseases, and healthy subjects. Talairach and Tournoux [28] suggested a transformation algorithm to a standard atlas involving the identification of various brain landmarks and piecemeal scaling of brain quadrants. An alternative approach was to use automated brain registration algorithms [29,30]. In the present study, a semiautomatic spatial normalisation utilizing a study specific template was performed for the transformation into MNI space. The template used for the normalisation of the DTI data sets was created from all subjects' (b = 0) data sets of the subjects who participated in this study. The iterative algorithm has previously been described in detail [31]. In short, a first template was generated by arithmetic averaging of the data sets after an affine transformation. Fitting of all data sets to this first template using a non-affine transformation and arithmetic averaging led to the template. Then, single subject DTI data sets could be normalised according to MNI dimensions.

Basically, a complete non-linear MNI normalisation consisted of 3 deformation components (DC):

• DC 1: A rigid brain transformation to align the basic coordinate frames. Hereby, the rotation angles had to be stored in a rotation matrix R→→
 MathType@MTEF@5@5@+=feaafiart1ev1aaatCvAUfKttLearuWrP9MDH5MBPbIqV92AaeXatLxBI9gBaebbnrfifHhDYfgasaacPC6xNi=xH8viVGI8Gi=hEeeu0xXdbba9frFj0xb9qqpG0dXdb9aspeI8k8fiI+fsY=rqGqVepae9pg0db9vqaiVgFr0xfr=xfr=xc9adbaqaaeGacaGaaiaabeqaaeqabiWaaaGcbaGafmOuaiLbaSGbaSaaaaa@2D23@.

• DC 2: An affine deformation according to landmarks. Hereby, the 6 stretching parameters for the different brain regions had to be stored in a 6-D vector S→
 MathType@MTEF@5@5@+=feaafiart1ev1aaatCvAUfKttLearuWrP9MDH5MBPbIqV92AaeXatLxBI9gBaebbnrfifHhDYfgasaacPC6xNi=xH8viVGI8Gi=hEeeu0xXdbba9frFj0xb9qqpG0dXdb9aspeI8k8fiI+fsY=rqGqVepae9pg0db9vqaiVgFr0xfr=xfr=xc9adbaqaaeGacaGaaiaabeqaaeqabiWaaaGcbaGafm4uamLbaSaaaaa@2D14@.

• DC 3: A non-affine normalisation equalizing non-linear brain shape differences. Hereby, the 3-D vector shifts were different for each voxel resulting in a 6-D matrix (a 3-D vector for each voxel of the 3-D matrix) T→→→
 MathType@MTEF@5@5@+=feaafiart1ev1aaatCvAUfKttLearuWrP9MDH5MBPbIqV92AaeXatLxBI9gBaebbnrfifHhDYfgasaacPC6xNi=xH8viVGI8Gi=hEeeu0xXdbba9frFj0xb9qqpG0dXdb9aspeI8k8fiI+fsY=rqGqVepae9pg0db9vqaiVgFr0xfr=xfr=xc9adbaqaaeGacaGaaiaabeqaaeqabiWaaaGcbaGafmivaqLbaSGbaSGbaSaaaaa@2D38@.

Consequently, the resulting diffusion tensor D→→i
 MathType@MTEF@5@5@+=feaafiart1ev1aaatCvAUfKttLearuWrP9MDH5MBPbIqV92AaeXatLxBI9gBaebbnrfifHhDYfgasaacPC6xNi=xH8viVGI8Gi=hEeeu0xXdbba9frFj0xb9qqpG0dXdb9aspeI8k8fiI+fsY=rqGqVepae9pg0db9vqaiVgFr0xfr=xfr=xc9adbaqaaeGacaGaaiaabeqaaeqabiWaaaGcbaGafmiraqKbaSGbaSaadaWgaaWcbaGaemyAaKgabeaaaaa@2E8E@ of each voxel *i *had to be rotated according to all the rotations listed above.

• Rotation resulting from the aligning to the basic coordinate frame (corresponding to DC 1)

D→→i'=R→→⋅D→→i.
 MathType@MTEF@5@5@+=feaafiart1ev1aaatCvAUfKttLearuWrP9MDH5MBPbIqV92AaeXatLxBI9gBaebbnrfifHhDYfgasaacPC6xNi=xI8qiVKYPFjYdHaVhbbf9v8qqaqFr0xc9vqFj0dXdbba91qpepeI8k8fiI+fsY=rqGqVepae9pg0db9vqaiVgFr0xfr=xfr=xc9adbaqaaeGacaGaaiaabeqaaeqabiWaaaGcbaGafmiraqKbaSGbaSaadaWgaaWcbaGaemyAaKgabeaakiabcEcaNiabg2da9iqbdkfaszaalyaalaGaeyyXICTafmiraqKbaSGbaSaadaWgaaWcbaGaemyAaKgabeaakiabc6caUaaa@3805@

• Simple trigonometry gives a rotation matrix (for each voxel independently), resulting from the 3-D vector shifts following the basic ideas of Alexander et al. [32]. The dilation matrices were used for the alignment of the tensor D→→
 MathType@MTEF@5@5@+=feaafiart1ev1aaatCvAUfKttLearuWrP9MDH5MBPbIqV92AaeXatLxBI9gBaebbnrfifHhDYfgasaacPC6xNi=xH8viVGI8Gi=hEeeu0xXdbba9frFj0xb9qqpG0dXdb9aspeI8k8fiI+fsY=rqGqVepae9pg0db9vqaiVgFr0xfr=xfr=xc9adbaqaaeGacaGaaiaabeqaaeqabiWaaaGcbaGafmiraqKbaSGbaSaaaaa@2D07@ of each voxel to the surrounding voxels (corresponding to DC 3).

D→→i''=t→→i⋅D→→i'
 MathType@MTEF@5@5@+=feaafiart1ev1aaatCvAUfKttLearuWrP9MDH5MBPbIqV92AaeXatLxBI9gBaebbnrfifHhDYfgasaacPC6xNi=xI8qiVKYPFjYdHaVhbbf9v8qqaqFr0xc9vqFj0dXdbba91qpepeI8k8fiI+fsY=rqGqVepae9pg0db9vqaiVgFr0xfr=xfr=xc9adbaqaaeGacaGaaiaabeqaaeqabiWaaaGcbaGafmiraqKbaSGbaSaadaWgaaWcbaGaemyAaKgabeaakiabcEcaNiabcEcaNiabg2da9iqbdsha0zaalyaalaWaaSbaaSqaaiabdMgaPbqabaGccqGHflY1cuWGebargaWcgaWcamaaBaaaleaacqWGPbqAaeqaaOGaei4jaCcaaa@3AA2@

where t→→i
 MathType@MTEF@5@5@+=feaafiart1ev1aaatCvAUfKttLearuWrP9MDH5MBPbIqV92AaeXatLxBI9gBaebbnrfifHhDYfgasaacPC6xNi=xH8viVGI8Gi=hEeeu0xXdbba9frFj0xb9qqpG0dXdb9aspeI8k8fiI+fsY=rqGqVepae9pg0db9vqaiVgFr0xfr=xfr=xc9adbaqaaeGacaGaaiaabeqaaeqabiWaaaGcbaGafmiDaqNbaSGbaSaadaWgaaWcbaGaemyAaKgabeaaaaa@2EEE@ are the components of T→→→
 MathType@MTEF@5@5@+=feaafiart1ev1aaatCvAUfKttLearuWrP9MDH5MBPbIqV92AaeXatLxBI9gBaebbnrfifHhDYfgasaacPC6xNi=xH8viVGI8Gi=hEeeu0xXdbba9frFj0xb9qqpG0dXdb9aspeI8k8fiI+fsY=rqGqVepae9pg0db9vqaiVgFr0xfr=xfr=xc9adbaqaaeGacaGaaiaabeqaaeqabiWaaaGcbaGafmivaqLbaSGbaSGbaSaaaaa@2D38@.

• The components of the Eigenvectors (v→1,v→2,v→3
 MathType@MTEF@5@5@+=feaafiart1ev1aaatCvAUfKttLearuWrP9MDH5MBPbIqV92AaeXatLxBI9gBaebbnrfifHhDYfgasaacPC6xNi=xH8viVGI8Gi=hEeeu0xXdbba9frFj0xb9qqpG0dXdb9aspeI8k8fiI+fsY=rqGqVepae9pg0db9vqaiVgFr0xfr=xfr=xc9adbaqaaeGacaGaaiaabeqaaeqabiWaaaGcbaGafmODayNbaSaadaWgaaWcbaGaeGymaedabeaakiabcYcaSiqbdAha2zaalaWaaSbaaSqaaiabikdaYaqabaGccqGGSaalcuWG2bGDgaWcamaaBaaaleaacqaIZaWmaeqaaaaa@3596@) had to be stretched according to the 6 stretching parameters of vector S→
 MathType@MTEF@5@5@+=feaafiart1ev1aaatCvAUfKttLearuWrP9MDH5MBPbIqV92AaeXatLxBI9gBaebbnrfifHhDYfgasaacPC6xNi=xH8viVGI8Gi=hEeeu0xXdbba9frFj0xb9qqpG0dXdb9aspeI8k8fiI+fsY=rqGqVepae9pg0db9vqaiVgFr0xfr=xfr=xc9adbaqaaeGacaGaaiaabeqaaeqabiWaaaGcbaGafm4uamLbaSaaaaa@2D14@ (dependent on the brain region *s*_*a*_, *a *= 1...6) of the affine deformation (corresponding to DC 2).

*v*_*w, j*_''' = *s*_*a *_*v*_*w, j*_'''

with *w *= 1...3 and *j *= *x*, *y*, *z*. After the stretching, the Eigenvectors had to be re-normalised.

These fine-corrections of the tensor D→→
 MathType@MTEF@5@5@+=feaafiart1ev1aaatCvAUfKttLearuWrP9MDH5MBPbIqV92AaeXatLxBI9gBaebbnrfifHhDYfgasaacPC6xNi=xH8viVGI8Gi=hEeeu0xXdbba9frFj0xb9qqpG0dXdb9aspeI8k8fiI+fsY=rqGqVepae9pg0db9vqaiVgFr0xfr=xfr=xc9adbaqaaeGacaGaaiaabeqaaeqabiWaaaGcbaGafmiraqKbaSGbaSaaaaa@2D07@ were essential for a correct FT (see section 4), and the corresponding parameters had to be stored for each subject data set independently. With the same normalisation procedure (without fine-corrections), the corresponding MPRAGE were normalised.

#### 2.2.4. Analysis methods – FA mapping and averaging of FA maps for different subjects

The second-rank diffusion tensor D→→
 MathType@MTEF@5@5@+=feaafiart1ev1aaatCvAUfKttLearuWrP9MDH5MBPbIqV92AaeXatLxBI9gBaebbnrfifHhDYfgasaacPC6xNi=xH8viVGI8Gi=hEeeu0xXdbba9frFj0xb9qqpG0dXdb9aspeI8k8fiI+fsY=rqGqVepae9pg0db9vqaiVgFr0xfr=xfr=xc9adbaqaaeGacaGaaiaabeqaaeqabiWaaaGcbaGafmiraqKbaSGbaSaaaaa@2D07@ could always be diagonalized, leaving only three non-zero elements along the main diagonal of the tensor, the Eigenvalues (*λ*_1_, *λ*_2_, *λ*_3_). The Eigenvalues reflected the shape or configuration of the ellipsoid. The diffusion anisotropy could be quantified by the fractional anisotropy:

Ff=(λ1−λ¯)2+(λ2−λ¯)2+(λ3−λ¯)2λ12+λ22+λ32
 MathType@MTEF@5@5@+=feaafiart1ev1aaatCvAUfKttLearuWrP9MDH5MBPbIqV92AaeXatLxBI9gBaebbnrfifHhDYfgasaacPC6xNi=xI8qiVKYPFjYdHaVhbbf9v8qqaqFr0xc9vqFj0dXdbba91qpepeI8k8fiI+fsY=rqGqVepae9pg0db9vqaiVgFr0xfr=xfr=xc9adbaqaaeGacaGaaiaabeqaaeqabiWaaaGcbaGaemOray0aaSbaaSqaaiabdAgaMbqabaGccqGH9aqpdaGcaaqaaKqbaoaalaaabaGaeiikaGccciGae83UdW2aaSbaaeaacqaIXaqmaeqaaiabgkHiTmaanaaabaGae83UdWgaaiabcMcaPmaaCaaabeqaaiabikdaYaaacqGHRaWkcqGGOaakcqWF7oaBdaWgaaqaaiabikdaYaqabaGaeyOeI0Yaa0aaaeaacqWF7oaBaaGaeiykaKYaaWbaaeqabaGaeGOmaidaaiabgUcaRiabcIcaOiab=T7aSnaaBaaabaGaeG4mamdabeaacqGHsisldaqdaaqaaiab=T7aSbaacqGGPaqkdaahaaqabeaacqaIYaGmaaaabaGae83UdW2aa0baaeaacqaIXaqmaeaacqaIYaGmaaGaey4kaSIae83UdW2aa0baaeaacqaIYaGmaeaacqaIYaGmaaGaey4kaSIae83UdW2aa0baaeaacqaIZaWmaeaacqaIYaGmaaaaaaWcbeaaaaa@57D2@

where λ¯
 MathType@MTEF@5@5@+=feaafiart1ev1aaatCvAUfKttLearuWrP9MDH5MBPbIqV92AaeXatLxBI9gBaebbnrfifHhDYfgasaacPC6xNi=xH8viVGI8Gi=hEeeu0xXdbba9frFj0xb9qqpG0dXdb9aspeI8k8fiI+fsY=rqGqVepae9pg0db9vqaiVgFr0xfr=xfr=xc9adbaqaaeGacaGaaiaabeqaaeqabiWaaaGcbaWaa0aaaeaaiiGacqWF7oaBaaaaaa@2D9F@ was the average of all Eigenvalues.

The intensity was related to the FA and the colour-coding was as follows: red for major Eigenvector mainly in left-right direction, blue for major Eigenvector mainly in inferior-superior direction and green for major Eigenvector mainly in posterior-anterior direction.

A ROI-based approach of defined brain regions was the method of choice for the analysis of separate areas. A ROI was defined by a sphere with an observer-defined radius *r*_*s *_and its centre at the user-defined focus f→s
 MathType@MTEF@5@5@+=feaafiart1ev1aaatCvAUfKttLearuWrP9MDH5MBPbIqV92AaeXatLxBI9gBaebbnrfifHhDYfgasaacPC6xNi=xH8viVGI8Gi=hEeeu0xXdbba9frFj0xb9qqpG0dXdb9aspeI8k8fiI+fsY=rqGqVepae9pg0db9vqaiVgFr0xfr=xfr=xc9adbaqaaeGacaGaaiaabeqaaeqabiWaaaGcbaGafmOzayMbaSaadaWgaaWcbaGaem4Camhabeaaaaa@2ED5@. All FA values *F*_*i *_of the *N *voxels inside this sphere (position v→i
 MathType@MTEF@5@5@+=feaafiart1ev1aaatCvAUfKttLearuWrP9MDH5MBPbIqV92AaeXatLxBI9gBaebbnrfifHhDYfgasaacPC6xNi=xH8viVGI8Gi=hEeeu0xXdbba9frFj0xb9qqpG0dXdb9aspeI8k8fiI+fsY=rqGqVepae9pg0db9vqaiVgFr0xfr=xfr=xc9adbaqaaeGacaGaaiaabeqaaeqabiWaaaGcbaGafmODayNbaSaadaWgaaWcbaGaemyAaKgabeaaaaa@2EE1@) were included in the following parameterisation. The ROI analysis included the following parameters:

• average FA, i.e. average diffusion strength

Favg=1N∑i,|f→s−v→i|<rsNFi
 MathType@MTEF@5@5@+=feaafiart1ev1aaatCvAUfKttLearuWrP9MDH5MBPbIqV92AaeXatLxBI9gBaebbnrfifHhDYfgasaacPC6xNi=xI8qiVKYPFjYdHaVhbbf9v8qqaqFr0xc9vqFj0dXdbba91qpepeI8k8fiI+fsY=rqGqVepae9pg0db9vqaiVgFr0xfr=xfr=xc9adbaqaaeGacaGaaiaabeqaaeqabiWaaaGcbaGaemOray0aaSbaaSqaaiabdggaHjabdAha2jabdEgaNbqabaGccqGH9aqpjuaGdaWcaaqaaiabigdaXaqaaiabd6eaobaadaaeWaqaaiabdAeagnaaBaaabaGaemyAaKgabeaaaeaacqWGPbqAcqGGSaaldaabdaqaaiqbdAgaMzaalaWaaSbaaeaacqWGZbWCaeqaaiabgkHiTiqbdAha2zaalaWaaSbaaeaacqWGPbqAaeqaaaGaay5bSlaawIa7aiabgYda8iabdkhaYnaaBaaabaGaem4CamhabeaaaeaacqWGobGtaiabggHiLdaaaa@4B21@

• standard deviation of averaged FA

Fstd=1N−1∑i,|f→s−v→i|<rsN(Favg−Fi)2
 MathType@MTEF@5@5@+=feaafiart1ev1aaatCvAUfKttLearuWrP9MDH5MBPbIqV92AaeXatLxBI9gBaebbnrfifHhDYfgasaacPC6xNi=xI8qiVKYPFjYdHaVhbbf9v8qqaqFr0xc9vqFj0dXdbba91qpepeI8k8fiI+fsY=rqGqVepae9pg0db9vqaiVgFr0xfr=xfr=xc9adbaqaaeGacaGaaiaabeqaaeqabiWaaaGcbaGaemOray0aaSbaaSqaaiabdohaZjabdsha0jabdsgaKbqabaGccqGH9aqpjuaGdaGcaaqaamaalaaabaGaeGymaedabaGaemOta4KaeyOeI0IaeGymaedaamaaqadabaWaaeWaaeaacqWGgbGrdaWgaaqaaiabdggaHjabdAha2jabdEgaNbqabaGaeyOeI0IaemOray0aaSbaaeaacqWGPbqAaeqaaaGaayjkaiaawMcaamaaCaaabeqaaiabikdaYaaaaeaacqWGPbqAcqGGSaaldaabdaqaaiqbdAgaMzaalaWaaSbaaeaacqWGZbWCaeqaaiabgkHiTiqbdAha2zaalaWaaSbaaeaacqWGPbqAaeqaaaGaay5bSlaawIa7aiabgYda8iabdkhaYnaaBaaabaGaem4CamhabeaaaeaacqWGobGtaiabggHiLdaabeaaaaa@55FF@

where *N *was the ROI size. Extensive studies via ROI analyses about the preservation of the DTI specific parameters during the process of MNI normalisation were performed previously [31].

Group studies might be of interest if the common deficit was due to damage of one or more defined brain areas. For this task, averaging of results for different subjects was necessary. After all subjects' data had been transformed into MNI space, averaging of the results was feasible. The latter step could be performed in three ways:

• The FA map was calculated separately for each subject data in MNI space, followed by arithmetic averaging of the FA maps. This led to a group-specific FA map FAM1. Hereby, the drawback was that the directional information was lost, quantification and analysis was performed via the FA maps.

• Diffusion tensor D→→
 MathType@MTEF@5@5@+=feaafiart1ev1aaatCvAUfKttLearuWrP9MDH5MBPbIqV92AaeXatLxBI9gBaebbnrfifHhDYfgasaacPC6xNi=xH8viVGI8Gi=hEeeu0xXdbba9frFj0xb9qqpG0dXdb9aspeI8k8fiI+fsY=rqGqVepae9pg0db9vqaiVgFr0xfr=xfr=xc9adbaqaaeGacaGaaiaabeqaaeqabiWaaaGcbaGafmiraqKbaSGbaSaaaaa@2D07@ matrices were stored after fine-correction and an averaged diffusion tensor matrix was created. This method required 600 Mbyte for each subject and increased computation time inadequately and thus was practicable only for studies with small populations.

• Each DTI data set was normalised and the whole DTI data sets were averaged before FA mapping. FA parameterisation of these group-averaged DTI data led to a group-specific FA map FAM2. The fine corrections were also arithmetically averaged. This resulted in an averaged fine correction to be applied to the resulting averaged DTI data set.

The third approach could be applied if the evidence that the FA values were preserved and also the orientational dependence of the Eigenvectors were preserved. ROI analysis had already shown that FA values were preserved by MNI transformation [31]. In section 4, a short excursion to computer simulation should give evidence that the orientational information could be preserved if fine corrections were applied.

#### 2.2.5. Fibre tracking

For FT, anisotropic diffusion was characterised to determine the preferred diffusion direction. In the calculation of the diffusion spheroid, the Eigenvector corresponding to the largest Eigenvalue was the direction of fastest diffusion and indicated the fibre direction in white matter regions. Based on this directional information, different methods and algorithms had been proposed to estimate white matter connectivity. In this work, the conservative streamline tracking technique (STT) was used. STT modelled the propagation in the major Eigenvector field of the brain [20,33].

Generally, the FT positions resulted from float numbers. The corresponding Eigenvector direction (to obtain the consecutive FT position) was the interpolation of the directions of the neighboured voxels weighted by the proportionate position (linear nearest neighbour interpolation)

v→new(i,j,k)=∑w=18awv→(lw,mw,nw)
 MathType@MTEF@5@5@+=feaafiart1ev1aaatCvAUfKttLearuWrP9MDH5MBPbIqV92AaeXatLxBI9gBaebbnrfifHhDYfgasaacPC6xNi=xI8qiVKYPFjYdHaVhbbf9v8qqaqFr0xc9vqFj0dXdbba91qpepeI8k8fiI+fsY=rqGqVepae9pg0db9vqaiVgFr0xfr=xfr=xc9adbaqaaeGacaGaaiaabeqaaeqabiWaaaGcbaGafmODayNbaSaadaWgaaWcbaGaemOBa4MaemyzauMaem4DaChabeaakiabcIcaOiabdMgaPjabcYcaSiabdQgaQjabcYcaSiabdUgaRjabcMcaPiabg2da9maaqadabaGaemyyae2aaSbaaSqaaiabdEha3bqabaGccuWG2bGDgaWcaiabcIcaOiabdYgaSnaaBaaaleaacqWG3bWDaeqaaOGaeiilaWIaemyBa02aaSbaaSqaaiabdEha3bqabaGccqGGSaalcqWGUbGBdaWgaaWcbaGaem4DaChabeaakiabcMcaPaWcbaGaem4DaCNaeyypa0JaeGymaedabaGaeGioaGdaniabggHiLdaaaa@522A@

Here, v→new(i,j,k)
 MathType@MTEF@5@5@+=feaafiart1ev1aaatCvAUfKttLearuWrP9MDH5MBPbIqV92AaeXatLxBI9gBaebbnrfifHhDYfgasaacPC6xNi=xH8viVGI8Gi=hEeeu0xXdbba9frFj0xb9qqpG0dXdb9aspeI8k8fiI+fsY=rqGqVepae9pg0db9vqaiVgFr0xfr=xfr=xc9adbaqaaeGacaGaaiaabeqaaeqabiWaaaGcbaGafmODayNbaSaadaWgaaWcbaGaemOBa4MaemyzauMaem4DaChabeaakiabcIcaOiabdMgaPjabcYcaSiabdQgaQjabcYcaSiabdUgaRjabcMcaPaaa@3948@ was the resulting vector at the new position *i*, *j*, *k *(float numbers), and *l*, *m*, *n *were the voxel coordinates (integer numbers) of the 8 neighboured voxels. The factors *a*_*w *_were the respective 8 weighting factors for the interpolation.

The following set of parameters was used for FT:

• The threshold for the scalar product of the major Eigenvectors (angle between directions of two consecutive FT positions and the first Eigenvector directions) was set to 0.95.

• The distance between two FT positions, i.e. the stepwidth, was set to 0.5 mm, corresponding to 0.5 voxels.

All the analyses were performed by the newly developed software package TIFT (Tensor Imaging and Fibre Tracking) [31]. TIFT provides various quantification and visualisation possibilities for DTI analysis. The structure of the software aims at minimisation of operator-dependency providing analysis in a fast and reproducible way.

#### 2.2.6. Tractwise fractional anisotropy statistics (TFAS)

Beside ROI analysis statistical comparison between subject groups could also be performed defining a skeleton which then was used for the selection of voxels that contribute to the statistics. FT was used to define such skeletons. When FT was performed on averaged DTI data, each voxel that is crossed by a FT was defined as 'active' for statistics. Basically, two skeletons could be built:

• A skeleton based on FT in averaged normal subject data (skeleton 1). The skeleton followed well known paths and was not disturbed by neuroanatomical alterations.

• A skeleton based on FT in averaged tCC subject data (skeleton 2). This skeleton was only under consideration for the sake of completeness.

In analogy to Equations 7 and 8, average FA and standard deviation were calculated:

Favg=1N∑iNFi,∀voxeli∈skeleton
 MathType@MTEF@5@5@+=feaafiart1ev1aaatCvAUfKttLearuWrP9MDH5MBPbIqV92AaeXatLxBI9gBaebbnrfifHhDYfgasaacPC6xNi=xI8qiVKYPFjYdHaVhbbf9v8qqaqFr0xc9vqFj0dXdbba91qpepeI8k8fiI+fsY=rqGqVepae9pg0db9vqaiVgFr0xfr=xfr=xc9adbaqaaeGacaGaaiaabeqaaeqabiWaaaGcbaqbaeqabeabaaaabaGaemOray0aaSbaaSqaaiabdggaHjabdAha2jabdEgaNbqabaGccqGH9aqpjuaGdaWcaaqaaiabigdaXaqaaiabd6eaobaadaaeWaqaaiabdAeagnaaBaaabaGaemyAaKgabeaaaeaacqWGPbqAaeaacqWGobGtaiabggHiLdGaeiilaWcakeaacqGHaiIiaeaacqWG2bGDcqWGVbWBcqWG4baEcqWGLbqzcqWGSbaBaeaacqWGPbqAcqGHiiIZcqWGZbWCcqWGRbWAcqWGLbqzcqWGSbaBcqWGLbqzcqWG0baDcqWGVbWBcqWGUbGBaaaaaa@52FC@

Fstd=1N−1∑iN(Favg−Fi)2,∀voxeli∈skeleton
 MathType@MTEF@5@5@+=feaafiart1ev1aaatCvAUfKttLearuWrP9MDH5MBPbIqV92AaeXatLxBI9gBaebbnrfifHhDYfgasaacPC6xNi=xI8qiVKYPFjYdHaVhbbf9v8qqaqFr0xc9vqFj0dXdbba91qpepeI8k8fiI+fsY=rqGqVepae9pg0db9vqaiVgFr0xfr=xfr=xc9adbaqaaeGacaGaaiaabeqaaeqabiWaaaGcbaqbaeqabeabaaaabaGaemOray0aaSbaaSqaaiabdohaZjabdsha0jabdsgaKbqabaGccqGH9aqpdaGcaaqaaKqbaoaalaaabaGaeGymaedabaGaemOta4KaeyOeI0IaeGymaedaamaaqadabaWaaeWaaeaacqWGgbGrdaWgaaqaaiabdggaHjabdAha2jabdEgaNbqabaGaeyOeI0IaemOray0aaSbaaeaacqWGPbqAaeqaaaGaayjkaiaawMcaamaaCaaabeqaaiabikdaYaaaaeaacqWGPbqAaeaacqWGobGtaiabggHiLdaaleqaaOGaeiilaWcabaGaeyiaIicabaGaemODayNaem4Ba8MaemiEaGNaemyzauMaemiBaWgabaGaemyAaKMaeyicI4Saem4CamNaem4AaSMaemyzauMaemiBaWMaemyzauMaemiDaqNaem4Ba8MaemOBa4gaaaaa@5DE5@

where *N *was the respective skeleton size, which usually was different for different skeletons. Then, TFAS used *F*_*avg *_and *F*_*std *_for statistical t-test.

#### 2.2.7 Statistical analysis

Statistical analysis (SA) by use of Student's t-test was performed in two ways:

SA1: skeleton 1 was applied to FA maps of the individual subjects. Alternatively, skeleton 2 was applied to FA maps of the individual subjects.

SA2: skeleton 1 and 2, respectively, were applied to the FA maps of the averaged DTI data sets. Hereby, the variability was calculated along the skeleton, so this approach is not an analysis of the group variability.

## 3. Results

### 3.1. Fibre track simulations

**Figure 1:**
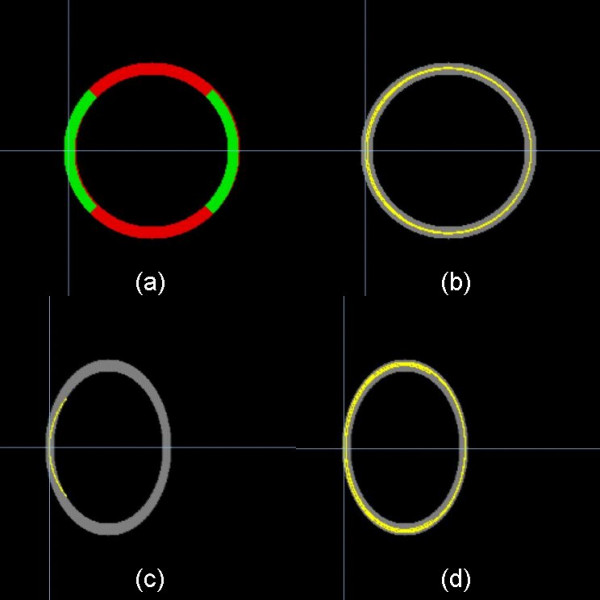
DTI simulations: **(a) **Circle simulation with inner radius of 64 pixels and outer radius of 74 pixels. **(b) **Corresponding fibre tracking (FT) results (light yellow). **(c) **Compression with a factor 1.4 destroys the FT (light yellow). **(d) **Correction of the Eigenvectors restored correct FT results (light yellow).

Since FT is known to be affected by MNI normalisations, the algorithms were tested on a 2-D simulation scenario consisting of a circle band with an inner radius of 64 voxels and an outer radius of 74 voxels. FA values were kept constant, and Eigenvectors pointed perpendicular to the radius vector (Figure 1a). Figure 1b shows FT results in the circle. In the second step, a deformation, i.e. a compression with a factor of 1.4, was performed (Figure 1c). If the diffusion tensor and the corresponding Eigenvectors were not corrected, FT failed (Figure 1c), whereas the correction of the Eigenvectors according to section 3.3 provided correct FT results (Figure 1d).

### 3.2. Differences between patients with tCC and controls

As an example of a disorder with a presumable affection of white matter tracts, patients with tCC were chosen. The CC was considered an important structure to investigate since a multitude of strongly directed fibres (connecting the hemispheres of the brain) is clustered in its formation. DTI should be able to detect axonal loss or at least damage of oriented white matter regions such as the CC.

#### 3.2.1. Fractional anisotropy mapping

**Figure 2:**
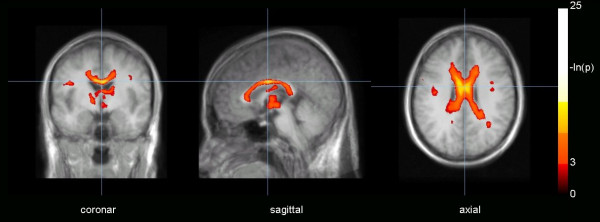
Whole brain-based spatial Students t-test on a voxelwise basis. Comparison of fractional anisotropy (FA) maps of 6 normal controls and 6 subjects with thinned corpus callosum (tCC). Display threshold was p < 0.05. The results were overlaid on an MPRAGE template (averaged from all 12 subjects who participated in the study).

Averaged FA maps were calculated with method FAM1. Whole brain based spatial statistics, i.e. voxelwise comparison of 6 FA maps from healthy controls and 6 FA maps of tCC patients by Students t-test resulted in Figure 2. Display p-value threshold was p < 0.05, significant differences were especially found in the CC. Five seeds in the CC were selected for the following analysis (Table 1). The seeds with high significance acted as centres for the following FT based TFAS analysis. ROI analysis of several regions in the CC gave the results listed in Table 2. Significant differences were found in 4 of the 5 seeds, whereas the ROI analysis of seed 5 was not significant.

**Table 1:** MNI coordinates of seeds in the corpus callosum (CC)

	x/mm	y/mm	z/mm
seed 1	0	23	8
seed 2	0	15	23
seed 3	0	-9	36
seed 4	0	-31	29
seed 5	0	-44	16

**Table 2:** Region of interest (ROI) analysis of fractional anisotropy (FA) maps with radius 5 mm at seeds in the corpus callosum (CC)

	FA (normals)	FA (tCC)	p
seed 1	0.61 ± 0.13	0.19 ± 0.04	<0.001
seed 2	0.40 ± 0.14	0.12 ± 0.01	0.02
seed 3	0.47 ± 0.13	0.13 ± 0.03	0.004
seed 4	0.43 ± 0.13	0.20 ± 0.04	0.06
seed 5	0.53 ± 0.13	0.48 ± 0.05	0.67

#### 3.2.2. Fibre tracking on single subjects

**Figure 3:**
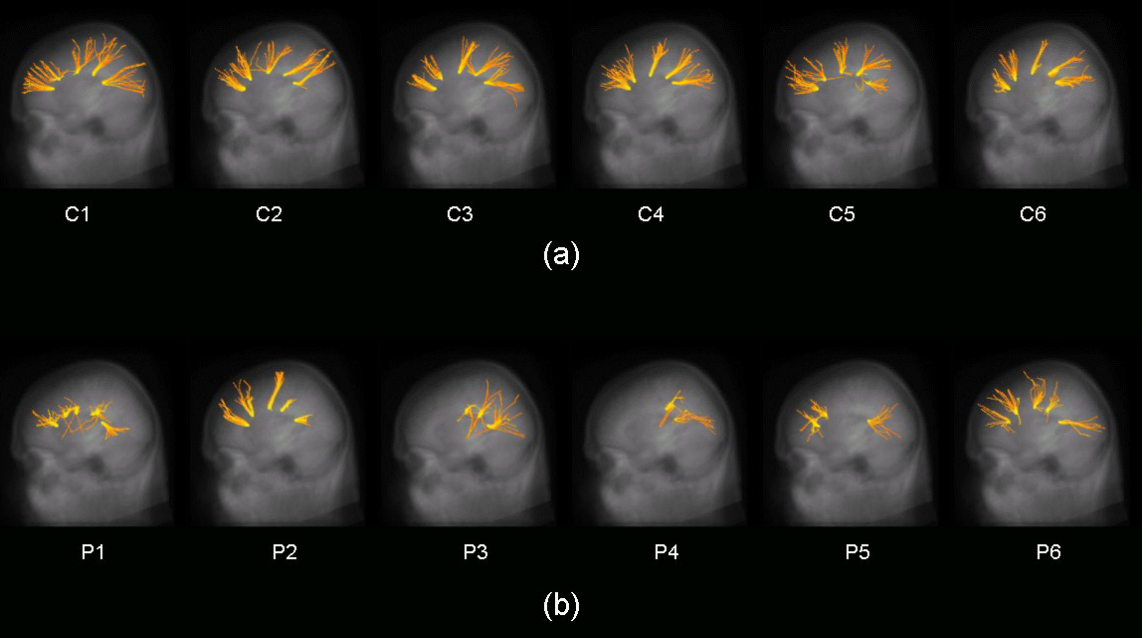
Fibre tracking (FT) with starting seeds in the corpus callosum (CC) (cf. **Table 1**): **(a) **6 healthy controls (C1 – C6). **(b) **6 subjects with thinned CC (tCC) (P1 – P6). The images are glass-brain plots of FT and the averaged MPRAGE data set. For the patients with tCC, not all possible seeds could be used for FT because the fractional anisotropy (FA) values were <0.05.

For comparison of the single subjects, FT was performed for all 12 subjects participating in the study (Figure 3). Healthy controls FT's looked very similar among each other and also similar to the FT from the averaged data set whereas in tCC patients FT failed due to a reduction of the averaged FA value at the ROI around the starting point of the FT below 0.05.

#### 3.2.3. Tractwise fractional anisotropy statistics

**Figure 4:**
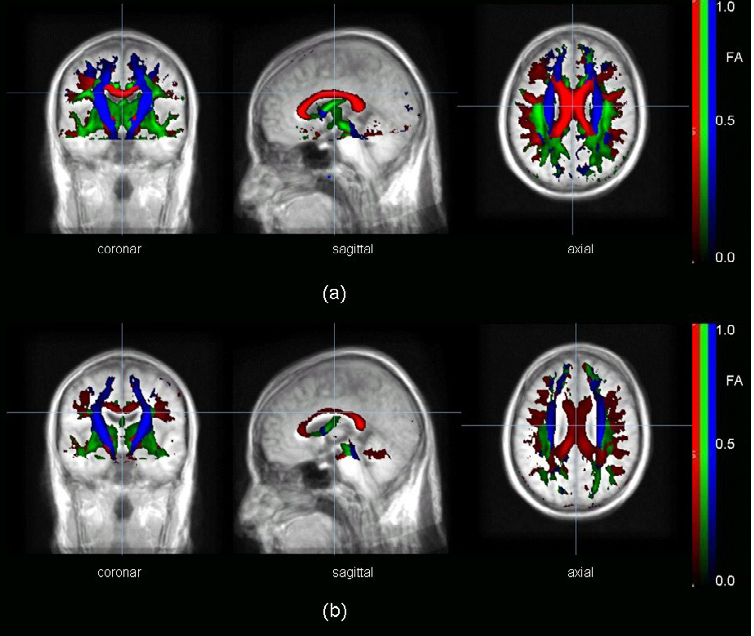
Fractional anisotropy (FA) maps for averaged data sets: **(a) **healthy controls, **(b) **thinned corpus callosum (tCC) subjects. Display threshold was FA > 0.2. The results were overlaid on an MPRAGE template (averaged from the 12 subjects participating in the study).

It was shown previously by ROI analyses that MNI normalisation preserves quantitatively FA values [31]. As demonstrated by the simulations in section 3.3, orientational dependencies of FT could also be preserved if the necessary fine-corrections were taken into account. Thus, if magnitude and orientation could be preserved, the averaging method FAM2 could be applied (Figure 4). Then, two averaged DTI data sets, one representing the 6 controls with normal CC structures and one representing the 6 tCC patients, were generated, and FT could be applied.

**Figure 5:**
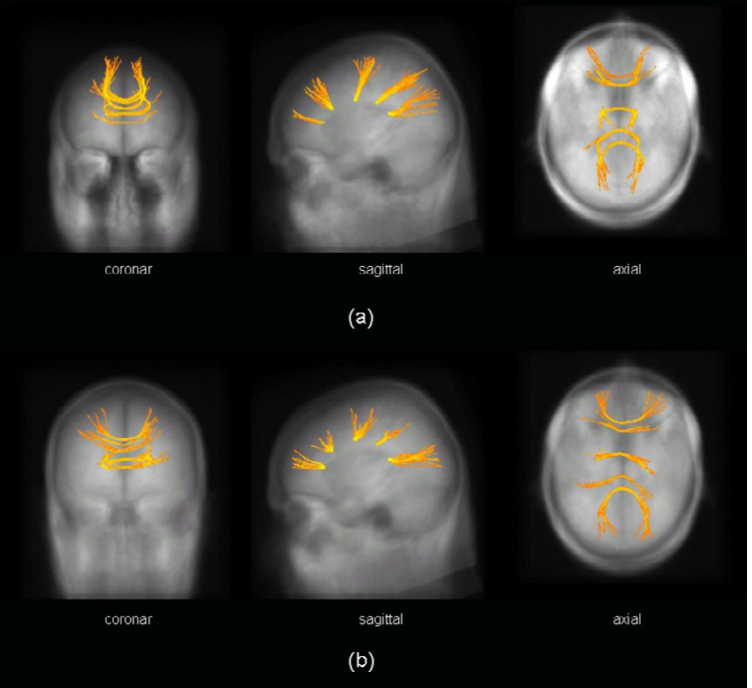
Fibre tracking (FT) with starting seeds in the corpus callosum (CC) (cf. **Table 1**): **(a) **averaged DTI data set from 6 normal subjects, **(b) **averaged DTI data set from 6 subjects with thinned CC (tCC). The images are glass-brain plots of FT overlaid on the averaged MPRAGE data set.

Taking the five seeds in the CC as starting points (following basically [17]), FT analysis led to results as displayed in Figure 5. FT results for the data set which represented the tCC patients (Figure 5b) showed a reduction compared to the data set which represented the healthy controls (Figure 5a). Therefore, several skeletons were used for the following TFAS analysis (see section 2.6.6)

• TFAS with skeleton 1: FT from healthy controls

• TFAS with skeleton 2: FT from tCC patients

When skeleton 1 was applied to FA maps of the individual subjects (SA1), averaged FA value for all healthy subjects was 0.34 ± 0.04 and averaged FA value for all tCC patients was significantly reduced to 0.25 ± 0.06 (p < 0.01) (Table 3, upper panel). By application of skeleton 2, results were very similar to the results for skeleton 1. Averaged FA value for all healthy subjects was 0.39 ± 0.03, averaged FA value for all tCC patients was significantly reduced to 0.26 ± 0.07 (p < 0.01) (Table 3, lower panel).

**Table 3:** Tractwise fractional anisotropy statistics (TFAS) with different skeletons, based on the 5 seeds in the corpus callosum (CC) (according to Table 1).

skeleton 1		
	FA (normals)	FA (tCC)

subject 1	0.37	0.19
subject 2	0.40	0.32
subject 3	0.32	0.22
subject 4	0.32	0.16
subject 5	0.31	0.31
subject 6	0.33	0.27
average	0.34 ± 0.04	0.25 ± 0.06

t-test: p < 0.01		

skeleton 2		

	FA (normals)	FA (tCC)

subject 1	0.40	0.20
subject 2	0.43	0.36
subject 3	0.40	0.22
subject 4	0.35	0.17
subject 5	0.36	0.31
subject 6	0.38	0.28
average	0.39 ± 0.03	0.26 ± 0.07

t-test: p < 0.01		

When skeleton 1 and 2, respectively, were applied to the FA maps of the averaged DTI data sets (SA2), TFAS showed a significant FA reduction independent of the skeleton applied. The averaged FA values for healthy controls were about 0.3 for all skeletons, and the averaged FA values for the tCC patients were significantly reduced to about 0.2 for all skeletons (p < 0.001). These results are listed in Table 4.

**Table 4:** Tractwise fractional anisotropy statistics (TFAS) with different skeletons, based on the 5 seeds in the corpus callosum (CC) (according to Table 1).

	FA (normals)	FA (tCC)	p
skeleton 1	0.28 ± 0.18	0.17 ± 0.09	<0.001
skeleton 2	0.33 ± 0.19	0.17 ± 0.09	<0.001

## 4. Discussion

With respect to the direction of fibres, the hypothesis could be set up that FT should give similar results for normals and patients with CC thinning. Nevertheless, it was shown that DTI and consecutive FT failed in some patients due to low FA values. Changes in orientational dependencies (FT) between the normal group and the tCC group were not related to changes in the diffusion direction; moreover, the low values in DTI (as detected by low FA values) led to errors in the diffusion tensors, and thus the orientational dependency was lost. To overcome this problem by improving the signal-to-noise ratio, averaging of DTI data from patients with a similar pattern of brain alterations was performed.

ROI analysis had already shown the preservation of FA values after MNI normalisation [31], whereas preservation of the orientational dependency during MNI normalization is feasible (as shown by computer simulations). Therefore, averaging of DTI data sets was considered to be allowed. Although the drawback of the averaging method (fine-corrections are averaged and thus could only partially be taken into account), the averaged DTI data set showed meaningful results for the FT. For the averaged data sets, the FT pattern looked more similar for controls and patients with tCC than the comparison of single subject data sets.

By application of tractography methods in order to obtain connectivity-based functional brain regions, as proposed by Behrens et al., region-specific FA analysis becomes feasible [6]. In addition to ROI- and whole brain-based analysis, this method takes the interconnectivity of brain regions into account. In the present study, TFAS was applied to skeletons which had been derived from different subject groups including patients with distorted brain anatomy. Here, the tractwise FA statistics again seemed to be superior to standard FA analysis methods due to its functional specifity.

It has to be kept in mind that averaging of DTI data sets was only possible if the deformations necessary for MNI normalisation were minor. Problems in cortical regions could occur as already reported by [32,31]. Although the FA values of the averaged DTI data sets were reduced by about 20 % due to the averaging process, the differences remain statistically significant for each of the statistical analysis methods.

## 5. Conclusion

In order to perform the analysis methods described in this study, a software platform was needed that was able to perform all the analysis at group level in a fast, reproducible and concise way, and the TIFT software contains all prerequisites for analysis performed in this work. At group level, the resulting averaged DTI data sets showed reduced FA maps as well as a reduction of FT for patients with tCC in comparison to healthy controls. Although the skeletons were different in localization and size, the result of a significant reduction of FA values, as calculated by TFAS in controls compared with tCC patients, was independent of the skeleton. Thus, TFAS seemed to be a probate way to measure differences at group level in groups of subjects with a groupwise similar pattern of brain alterations.
